# Efficacy of a prewriting intervention: A pilot randomised control trial

**DOI:** 10.1111/1440-1630.70043

**Published:** 2025-08-12

**Authors:** Berenice Johnston, Brooke Ryan, Megan Hatfield, Samuel D. Calder, Mary Claessen

**Affiliations:** ^1^ School of Allied Health Curtin University Perth Western Australia Australia; ^2^ Health Sciences, College of Health and Medicine University of Tasmania Launceston Tasmania Australia; ^3^ Professional Standards Team Speech Pathology Australia Melbourne Victoria Australia

**Keywords:** children, handwriting, occupational therapy, prewriting, schools, teachers

## Abstract

**Introduction:**

Successful handwriting is dependent on accurate and efficient letter formation, which is dependent on drawing sub‐strokes of letters and prewriting patterns. Currently, there is no prewriting intervention programmes with established efficacy, and little is known about children's perceptions of engaging in these programmes. This study aimed to determine the efficacy and feasibility of a prewriting intervention.

**Methods:**

A pilot randomised control trial was conducted with embedded aspects of fidelity and acceptability. Participants included 18 typically developing 4‐ to 5‐year‐old children, attending a Western Australian kindergarten (year before first formal schooling year), randomly allocated to an intervention or waitlist control group. Baseline and post intervention data were collected using the Developmental Test of Visual Perception (Third Edition) and the Prewriting Assessment (PWA). Participants received six Peggy Lego intervention sessions, and a fidelity checklist was completed following each session. Immediately following completion of the intervention, participants provided acceptability feedback using a modified Likert scale.

**Consumer and Community Involvement:**

Teachers and occupational therapists working with 4‐ to 5‐year‐old children provided feedback on the intervention.

**Results:**

There was a statistically significant main effect of time on the PWA score (*p* = 0.003); however, the main effect of group and the interaction of group and time were non‐significant (*p* = 0.070 and *p* = 0.46). The intervention was implemented with high levels of fidelity with 19 sessions (*n* = 34) achieving 100% fidelity. Eligibility was deemed feasible with 60% of those enrolled for the study meeting eligibility criteria. Most participants (*n* = 17) completed six intervention sessions. Most participants found the intervention acceptable (*n* = 13).

**Conclusions:**

Exploratory analysis showed all children significantly improved their prewriting ability; however, it is likely that this effect is not attributed to intervention alone. This pilot randomised control trial is deemed feasible in terms of recruitment, retention of participants, and data collection. Further research on the efficacy of this intervention is justified.

**PLAIN LANGUAGE SUMMARY:**

For writing to be easy to read, students need to form letters the right way and with good control. Before they can do this, they need to learn basic pencil strokes used to make letters. Handwriting programmes are used in schools and with children who find writing hard. We know some of these programmes help, but we do not know which prewriting programmes work best. We wanted to find out if a programme called Peggy Lego helps and if it is easy to use. We did six Peggy Lego sessions with 18 kindergarten children in Western Australia. All of the children got better at their prewriting skills, but we could not tell if Peggy Lego helped more than routine teaching, or if the children improved over time. We asked the students how they felt about the programme. Most said they liked it and thought their drawing got better, even though it was a bit hard. Future research could look at whether feeling more confident helps children when they start learning to write letters.

**Key Points for Occupational Therapy**

- Prewriting ability of 4 to 5‐year‐old children may improve naturally over time.
- Intervention did not lead to greater improvements compared to a waitlist control.
- Students enjoyed the intervention and felt their drawing skills improved.
- Further research is needed to determine the efficacy of Peggy Lego.

## 1 INTRODUCTION

The ability to efficiently and effectively communicate ideas and thoughts in writing is a critical skill required across many occupations of life, including productivity, daily living, relationships, and leisure. Handwriting, an important part of written expression, is acknowledged as a complex task, encompassing the integration of component level skills of visual–motor integration, in‐hand manipulation, sensory processing, motor planning, and cognitive and perceptual skills (Dinehart, 2015; Feder & Majnemer, 2007). As this task of handwriting becomes automatic, writing becomes fluent and less of a cognitive burden. The journey to reaching handwriting automaticity however starts with prewriting skills, even before a child commences formal schooling.

Prewriting is a term that encompasses performance components that contribute to learning to write (such as visual motor integration and in‐hand manipulation) (Asher, 2006). Prewriting patterns refer to the pencil strokes a child learns that are later used to form letters. These substrokes of letters include vertical lines, horizontal lines, circles, and diagonal lines, which translate directly into letter formation (Mathwin et al., 2022), for example, a vertical line is used to form the letter ‘l’ and a circle is used to form the letter ‘o’, and diagonal lines are used to form the letter ‘x’. Prewriting skills are a critical part of the early stages of handwriting and are recognised as developmental, where mastery of prerequisite skills is essential for future handwriting automaticity (Klein et al., 2021a).

There are strong links between handwriting and academic achievement, and some research indicates that the origin of this may occur in the years before formal schooling (Dinehart, 2015). Additionally, the relationship between handwriting and reading ability is well established (James & Engelhardt, 2012; Longcamp et al., 2005), as the motor act of handwriting assists a student to consolidate their letter recognition and phonemic awareness (two predictors of reading; Fitzpatrick et al., 2013). Effective handwriting is also linked with orthographic knowledge (van Galen, 1991) as the process of writing a letter not only involves motor systems but a linguistic process to convert the sound of the letter to the visual representation of the letter (phoneme to grapheme) (van Galen, 1991). This highlights that handwriting relies on several different cognitive and motor systems (Mathwin et al., 2022). Consequently, handwriting is recognised as a key component of literacy (Limpo & Graham, 2020) as well as academic success (Dinehart, 2015).

### 1.1 Importance of prewriting patterns in future formation of letters

As a student's ability to understand prewriting strokes precedes letter identification and letter‐forming skills, the ability to form prewriting patterns plays an important role in the developmental process of acquiring handwriting and the broader domain of written expression. Assumptions are often made, however, regarding a student's ability to produce prewriting patterns (Reutzel et al., 2019), and as such, a greater emphasis is needed on prewriting within both classrooms and research. A recent survey identified that within Australian kindergartens, around 25% of teachers did not include explicit prewriting instruction in their classroom teaching practice, which confirms Reutzel's findings (Johnston, Hatfield, et al., 2025). As the ability to form prewriting patterns is a prerequisite for handwriting function, it is essential that therapists and educators ensure they support the mastery of prewriting in the early years (Klein et al., 2021a), to support future success in written expression.

There are ‘rules’ relating to the way a letter is formed, specifically in relation to the motor action a person uses to form the letter, including the starting point of the letter, direction taken and end point of the letter (Mathwin et al., 2022). Additional quality factors impact on legibility of the letter, such as angles of the letter and smoothness of the pencil stroke. When a letter is correctly formed, the student recalls the motor plan for each individual letter with efficiency which in turn supports the development of handwriting automaticity (Mathwin et al., 2022). As letters are formed using a combination of prewriting patterns, the automatic recall of prewriting patterns may be required prior to the integration of these patterns into letter formations (Reutzel et al., 2019). Consequently, the correct formation of prewriting patterns within the kindergarten environment may directly impact on future letter formation ability (Klein et al., 2021a).

### 1.2 Prewriting challenges

Many students (up to 25–33%) experience challenges with handwriting (Benning et al., 2018). As 42% of a kindergarten student's day is spent on pencil and paper tasks (Marr et al., 2003), this difficulty has a significant impact on the child's classroom participation throughout the day. Challenges with handwriting is identified as one of the most common sources of referral for occupational therapy intervention for school‐aged children (Kadar et al., 2020); however, it is likely that a student's handwriting challenges arose in the prewriting stage of handwriting development. Currently, there is no evidence indicating the rate of difficulty for prewriting ability within the kindergarten year.

Children who experience handwriting challenges encounter negative impacts on their schooling and barriers with their classroom engagement (Bonneton‐Botté et al., 2023). When the barrier is significant, students may start avoiding the writing task or not engage in their instruction as the challenge they face is too large (Dinehart, 2015). As such, it is imperative to implement programmes that are both effective and acceptable to students, in order to ensure high levels of engagement in handwriting instruction. It is also essential that these programmes are implemented with fidelity to increase the confidence that the intended outcomes are achieved.

### 1.3 Current prewriting interventions

While there are programmes available to remediate challenges with handwriting function, there are limited evidence‐based programmes available that allow educators and therapists to support the development of prewriting function (Klein et al., 2021b). A systematic review of handwriting interventions for 4‐ to 6‐year olds (Kadar et al., 2020) identified that while intervention and occupational therapy consultation for teachers on handwriting can be beneficial, no high‐level studies were identified. Programmes referenced to support handwriting within this systematic review included Handwriting without Tears, Write Start, Steps K, and Size Matters (Kadar et al., 2020); however, they do not all include direct instruction on the prewriting patterns. Other studies have focussed on the component level skills that support handwriting development, such as postural and hand control, with limited instruction on prewriting patterns (Donica et al., 2013). There are conflicting views on the approach to learning prewriting; however, it has been indicated by some researchers that universal direct instruction of prewriting ensures all children learn the skills required which limits learning negative handwriting habits that can be hard to remediate, as well as the potential for future failure (Klein et al., 2021b).

Survey findings on prewriting instruction in Australian kindergartens indicated that numerous programmes are being used in classrooms, including the Peggy Lego programme, Preventing Literacy Difficulties, Writing in the Sand, Printing Like a Pro, Handwriting without Tears and a combination of programmes (*n* = 14), including Casey Caterpillar, Montessori prewriting skills, and Fantastic Fingers (Johnston, Hatfield, et al., 2025). This survey also identified that when teachers use a formal prewriting programme, they are more confident in their prewriting instruction. A challenge exists for both classroom teachers and occupational therapists regarding prewriting intervention; however, as while these programmes may be available for use in the classroom, there is limited empirical validation studies investigated the efficacy of how these programmes teach prewriting patterns (Klein et al., 2021b), particularly within the context of the Australian classroom (Kadar et al., 2020).

### 1.4 Theory to guide intervention

Considering evidence‐based approaches to handwriting instruction, Motor Learning Theory is applied to instruction (Klein et al., 2021a). Motor Learning Theory assists occupational therapists and teachers to understand how an individual learns the motor task of handwriting, describing the acquisition of motor skills according to three phases: cognitive, associative, and autonomous phase. Within the cognitive phase of learning how to handwrite, the student first learns the motor actions relating to the letter formations and dedicates a great deal of cognitive power to perform this novel motor task. This results in motor actions or letter formations that are slow and inconsistent. The associative phase, however, sees motor actions, or letter formations, become more efficient with less cognitive effort required to complete the motor task. The final phase is when the motor task, forming the letter, is efficient and effective, with little cognitive focus required (Zwicker & Harris, 2009). Instruction based on Motor Learning Theory also advocates for the grading of prompts according to the stage of learning (direct to indirect and external to internal). When this theory is applied to the motor task of handwriting, the development of handwriting progresses through these phases, with the aim for the student's handwriting to become an automatic motor task, requiring little additional cognitive effort. Instruction of letter formation as well as prewriting patterns should align with Motor Learning Theory to support efficient and fluent handwriting, so students are best supported to achieve the goal of handwriting automaticity (Klein et al., 2021a).

A prewriting and letter formation instruction programme that aligns with Motor Learning Theory is the Peggy Lego Programme (Lego, 1983). Designed by a Western Australian teacher in the 1980s, the Peggy Lego programme has two phases. The first phase teaches the formation prewriting patterns, in isolation to the concept of letters or sounds. The second phase explicitly teaches how prewriting patterns are embedded into letter formations, usually addressed in the first year of compulsory full‐time schooling. This developmentally staged approach to handwriting instruction is based on the concept that teaching letter formations, which are complex motor patterns, is easier to master as the prewriting patterns are understood and automatic to the student. This then allows for the prewriting pattern to be translated into letter formations with greater ease. While initially designed for use by teachers in classrooms (Lego, 1983), the Peggy Lego programme is also used by occupational therapists to remediate prewriting and letter formation challenges. The design of the programme aligns with Motor Learning Theory as it explicitly teaches the motor action required for each prewriting pattern, following a systematic approach to teaching letter formation moving students through the cognitive, associative, and autonomic phases of motor learning, grading of the level of prompting required. Within the Peggy Lego programme, instruction of the prewriting Peggy Lego patterns (seven in total) incorporates the systematic use of whole‐body movements imitating the prewriting pattern, tactile tasks where the student forms the prewriting pattern with their hands and fingers in a sensory activity and writing tool tasks where they form the prewriting pattern with a writing tool. Once these Peggy Lego patterns are mastered, students learn how to apply these patterns into picture drawings or letter formations. While the Peggy Lego programme has been identified as a commonly used programme within Australian kindergartens (Johnston, Hatfield, et al., 2025), there has been limited research on this programme. In the absence of evidence, we sought to justify the common use of the initial phase of the Peggy Lego programme in classrooms.

### 1.5 Current Australian classroom context

There is no national Australian English curriculum for kindergarten; however, the Western Australian School Curriculum and Standards Authority provides overarching kindergarten curriculum guidelines to guide classroom practice (School Curriculum and Standards Authority, 2023). Within this guideline, it references that students will engage in writing behaviours, including ‘exploring mark‐making using drawing, symbols, and familiar letters’ (School Curriculum and Standards Authority, 2023). The national English Curriculum for the foundation year of school, the year following kindergarten in Western Australia, then requires students to correctly form most upper and lower‐case letters (Australian Curriculum, Assessment and Reporting Authority, n.d.).

Within education, the Response to Intervention Model guides classroom instruction, identification of need and intervention (Fletcher & Vaughn, 2009). Within this model, there are three tiers, with tier one including core instructional interventions applied to all students in a preventative and proactive manner. Tier two involves targeted group interventions for some students where there is a focus on high efficiency and a rapid response to intervention. Tier three includes individual and intensive interventions that are assessment based and of longer duration. The term intervention is used across all tiers of this model.

### 1.6 Acceptability and fidelity

The measure of efficacy can confirm if a prewriting intervention works as intended; however, when evaluating interventions, it is important to consider not only the efficacy but feasibility elements of acceptability and fidelity. The acceptability of an intervention reflects how well participants receive the intervention and incorporates affective attitude of the recipient, the burden of the intervention, ethicality, intervention coherence, opportunity costs, perceived effectiveness and self‐efficacy (Sekhon et al., 2017). Fidelity ensures that the programme is delivered as designed (Borrelli et al., 2005), which helps to demonstrate the overall success and sustainability of any intervention.

While existing theoretical frameworks of acceptability exist to guide research design, there is limited evidence available regarding the application of this framework to intervention designed for children (Eckert et al., 2017), and no evidence could be sourced regarding the acceptability of a prewriting or handwriting intervention. Questionnaires are reported as a commonly used tool in gathering acceptability data where the participants provide feedback on the intervention using a Likert‐type scale (Finn & Sladeczek, 2001); however, different modalities are used, including individual and group interviews, formal acceptability scales, and research developed instruments (Eckert et al., 2017). Because of developmental factors, child responses and perspectives are impacted by cognition and comprehension of questions (Mellor & Moore, 2014) presenting a potential barrier to the collection of acceptability data. Factors relating to the power in the interviewer/interviewee relationship also need to be considered as children may feel obliged to provide a response that they perceive to be the correct answer (Lundy et al., 2011; Ponizovsky‐Bergelson et al., 2019). Understanding the acceptability of a prewriting intervention from a child's perspective is important as it can indicate the likelihood of intervention outcomes being achieved and indicate the potential effectiveness of the intervention (Eckhert et al., 2021).

When the fidelity of an intervention is assessed, it can assist in the confidence that the outcome of the intervention is a result of the intervention that has been implemented (Borrelli, 2011). Regarding prewriting interventions, even though teachers report higher levels of confidence in prewriting instruction when using a formal programme (Johnston, Hatfield, et al., 2025), the degree of fidelity with which prewriting programmes are implemented is unknown.

This study aims to address the existing gap in evidence relating to prewriting instruction by determining the feasibility and preliminary efficacy of a prewriting intervention, as well as the perceived student acceptability of the intervention and the fidelity with which it can be implemented. This study aligns with phase two of Robey's phases of study design (Robey, 2004), where the aim is to explore the dimensions of treatment effect to prepare for a potential clinical trial. Before this study could be expanded to see if there is established efficacy, this level of research was necessary.

Occupational therapists can play a key role in supporting teachers prewriting instruction and develop interventions that support young children in their prewriting: a critical component of occupational performance at school.

### 1.7 Research Aim and objectives

This study aimed to determine the feasibility and preliminary efficacy and of a prewriting intervention, the fidelity with which it is implemented, and if it was acceptable to the participants. These aims were guided by the following research objectives in relation to a 6‐week Peggy Lego intervention, implemented by an occupational therapist with 4 to 5‐year‐old children:

1. To determine the feasibility of implementing the intervention
2. To determine the fidelity of the intervention
3. To determine the level of acceptability of the intervention
4. To determine if the intervention has a treatment effect in the ability to draw prewriting patterns (as measured by the Prewriting Assessment (PWA]).

## 2 METHOD

### 2.1 Positionality

With professional backgrounds in occupational therapy and speech pathology, all authors share expertise in early literacy intervention and supporting children to engage and participate in Australian classrooms. Berenice Johnston, an experienced occupational therapist, has extensive experience in assessment and intervention of handwriting in children.

### 2.2 Design

#### 2.2.1 Efficacy

A crossover pilot randomised control trial research design with embedded aspects of fidelity and acceptability was used for this study, with a total of 18 participants from a Western Australian kindergarten. The participants were assessed using the Visual Motor Integration (VMI) sub‐tests of the Developmental Test of Visual Perception – Third Edition (DTVP [3]), copying (CO) and eye–hand coordination (EHC) and the PWA (12 pictures) (PWA [12]). Once deemed eligible to participate in the study, the participants were allocated to one of three intervention groups or three waitlist control groups, with three participants in each group.

#### 2.2.2 Feasibility and acceptability

Feasibility indicators including recruitment success, retention of participants, completeness of data collection, intervention fidelity and participant acceptability were considered throughout the study. A fidelity checklist was completed by the lead researcher following every intervention session, and a survey using a modified Likert scale was completed by participants to rate the acceptability of the intervention, facilitated by an independent occupational therapist. To determine interrater reliability, 20% of the assessments were rescored along with 20% of all fidelity checklists and acceptability surveys. Ethics approval was granted from Curtin University Human Research Ethics Committee.

### 2.3 Participants

A mainstream school within Catholic Education Western Australia was recruited for this study in May 2023. Study information was provided to the school principal by the lead researcher, who consented and confirmed the school met the eligibility criteria regarding the class‐based instruction of prewriting (could not be implementing the Peggy Lego programme). The school principal provided recruitment information to the three kindergarten class teachers, indicating the eligibility criteria for children to participate in the study. The kindergarten teachers identified students from their class based solely on the study's inclusion and exclusion criteria and disseminated participant information and consent forms on behalf of the researcher. Inclusion criteria included having English as their primary language and a date of birth prior to 27 April 2018. Exclusion criteria for the study included a diagnosis of Attention Deficit Hyperactivity Disorder, Autism Spectrum Disorder, Intellectual Disability, Developmental Language Disorder, or a neuro muscular disorder. Students receiving additional teacher support in the classroom or seeing a health professional including an occupational therapist, speech pathologist, physiotherapist, or paediatrician were excluded from the study. This information was detailed on the consent form where parents (or caregivers) answered questions about their child, confirming if their child met the inclusion criteria. Children were required to provide written assent to engage in the study by circling a green tick or red cross on an additional form, with an attempt to mark their name. Returned consent forms were reviewed by researcher to ensure eligibility criteria were met prior to the participant proceeding to the next stage of the study.

Once initial eligibility was confirmed, the participants were then assessed using the CO and EHC subtests of the DTVP (3) and PWA (12) to determine a second stage of eligibility. This second stage of eligibility required participants to score a minimum VMI composite index of 85 on the DTVP (3) and a score no higher than 50% on the PWA (12). A composite index of 85 was selected as the minimum requirement (i.e. 1 SD below the mean SEM = 4), to ensure participants had average visual motor skills. A ceiling score of 50% was selected on the PWA (12) to ensure participants who had already developed skills in forming prewriting patterns did not engage in unnecessary intervention, and we included those who still had not mastered skills in forming prewriting patterns.

As this study aimed to determine the feasibility of a future efficacy trial, no formal sample size calculations occurred.

### 2.4 Intervention

The group intervention sessions for this study were designed using the Peggy Lego programme and facilitated by an occupational therapist. The first phase of this programme explicitly teaches prewriting patterns, with either one or two new patterns introduced in each treatment session. The participants produce the pattern with their while body, hands, and fingers, as well as writing tools of chalk, markers, and crayons a minimum of 20 times per session. Visual aids, verbal prompts, and modelling of the patterns were also used by the researcher, and participants were encouraged to chant the verbal prompt that matched the prewriting pattern as they produced it. The format of the session, including the structure of activities, the wording for the researcher, and the timing of each section was detailed in an intervention protocol. The participants engaged in six weekly group intervention sessions running between 23 and 30 min each, on site at the school in a space the children were familiar with, away from their classroom, with three participants in each group. Treatment sessions were video recorded to support the completion of the fidelity checklist. See Table 1 for intervention and measures timeline. See Table 2 for Template for Intervention Description and Replication.

**TABLE 1:** Intervention and measures timeline.

Group	Term 2 week		Term 3 week						Term 4 week							
	4	7	5	6	7	8	9	10	1	2	3	4	5	6	7	8
Group 1 Intervention	R	BL1	I F	I F	I F	I F	I F	I F AC	PID							
Group 2 Waitlist Control	R	BL1							BL2	IF	I F	I F	I F	I F	I F	PID AC

*Note: R* = recruitment, BL 1 = baseline session one, including DTVP(3) and PWA(12); BL2 = baseline session two, including DTVP(3) and PWA(12); I = intervention session using Peggy Lego programme; F = fidelity checklist; AC = acceptability interview; and PID = post intervention data collection, including DTVP(3) and PWA(12).

Term refers to the term of the school year. In Western Australia, there are four school terms across the school year, commencing in February with term 1. Week refers to the week number within the school term. Most school terms in Western Australia consist of 10 weeks (± a week).

**TABLE 2:** *Template for intervention description and replication (TIDieR)*.

TIDieR item	Description
1.Brief name:	Peggy Lego prewriting intervention
2. Why:	Peggy Lego is a prewriting instruction programme that explicitly teaches the prewriting patterns required to form letters of the alphabet. The intervention aligns with motor learning theory which is known to support handwriting instruction up to 7 years of age The Peggy Lego programme uses whole body movement, tactile experiences verbal and visual cues to support the participant to learn the motor movement required to form the prewriting pattern, prior to them using a drawing tool to form the pattern. The first phase of the programme teaches the prewriting patterns in isolation to the concept of letters and sounds.
3. What:	Printed intervention protocol, visual posters of the prewriting patterns, long stretchy rope/theratubing, rainbow coloured ribbons, table and chairs, dry rice trays, chalk boards, chalk
Materials	Whiteboards, whiteboard markers, crayons, large sheets of blank paper, device for recording, tripod, floor space
4. What: procedures:	Within each small group session, each participant received instruction via an occupational therapist on one or two new prewriting patterns. Participants were taught the name of the shape, the accompanying verbal rhyme and shown a picture of the pattern. During the intervention, participants produced the prewriting pattern with their whole body, hands and fingers (in dry rice) and with a white board maker, chalk, and crayon tool. On completion of each session, a review was completed on all patterns addressed throughout all intervention sessions.
5. Who provided:	The intervention sessions were provided by an experienced registered occupational therapist (lead researcher), with experience in handwriting intervention in the school‐based setting. Pre and post data collection was completed by an independent registered occupational therapist blinded to the aims of the research. This occupational therapist was skilled in the administration of the DTVP (3) and trained in the administration and scoring of the PWA (12)
6. How:	Face to face small group sessions (three participants per group)
7. Where:	Intervention was provided on site at the participants school, in a quiet room familiar to them. Data collection was also completed in this room, or within a small room near their regular classroom.
8. When and how much:	The intervention was delivered over 6 × 30 min small group treatment sessions over a consecutive period of 6 weeks. Within each session participants repeated the prewriting pattern 20–30 times with their whole body, hands, and fingers or writing tool.
8. Tailoring:	Additional supports and prompts were provided to two participants to support their engagement in the treatment sessions.
9. Modifications:	Because of participant absence across five sessions, modifications were made providing individual make up sessions for three of the four students affected.
10. How well:	Treatment sessions were video recorded to support fidelity of the intervention. Following each intervention session, a fidelity checklist was completed by the lead researcher who facilitated the intervention. This fidelity checklist was rescored by an independent rater to allow for inter‐rater reliability to be determined.
Planned	Data collection of the PWA (12) were video rescored to allow for an independent rater to rescored the assessment to allow for inter‐rater reliability to be determined.
11. How well: Actual	High levels of fidelity were achieved in this pilot randomised control trial with 19 sessions achieving 100% fidelity (*n* = 34)

### 2.5 Outcome measures

The VMI subtests of the DTVP (Third Edition); CO, and EHC, along with the PWA, were used to confirm that participants met eligibility criteria. The PWA was also used as an outcome measure.

#### 2.5.1 DTVP – Third Edition

The DTVP (3) is a norm‐referenced assessment that provides information on the visual perception skills of the participant (Brown, 2016); however, through independent administration of the EHC and CO subtest, an overall VMI score is obtained. This measure was selected as VMI has been specifically identified as a predictor of handwriting success (Daly et al., 2003; Dinehart, 2015). Good test–retest reliability (between 0.70 and 0.90), robust internal consistency (between 0.80 and 0.95), and strong inter‐rater reliability (0.90) have been established for the DTVP (3) (Brown, 2016).

#### 2.5.2 PWA

**FIGURE 1:**
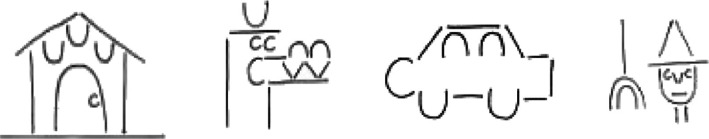
*Sample assessment test items pictures*.

A modified version of the PWA was used in this study, the PWA (12 pictures) (PWA [12]). The PWA requires children to use a pencil to copy eight pictures which are constructed of prewriting patterns onto a piece of A5 paper. See Figure 1 for sample assessment item. Scoring occurs according to two factors; the direction used to form the prewriting pattern within the picture and the quality of the prewriting pattern they produce. The directionality and quality factors for each picture are combined to generate a total test score, which is then converted to a percentage of success. The pictures within the PWA are not intended to increase in complexity as the assessment progresses, and all pictures presented within the assessment are required to be attempted. Currently, there is no ceiling score that requires the administration of the assessment to cease. The PWA has established reliability (0.82 for test re‐test reliability, 0.92 for inter‐rater reliability, and 0.97 for intra‐rater reliability [*p* < 0.001]) and criterion (concurrent) validity (DTVP [3] VMI sum of scaled scores r[52] = 0.58, *p* < 0.001), and excellent internal consistency (Johnston, Ryan, et al., 2025).

To minimise the risk of practise effects in this study, an expanded version of the PWA was created; the PWA (12) which included four additional pictures. As the directionality scoring of the PWA (12) requires the assessor to closely view the movement a student used when drawing the images, the administration of the PWA (12) was video recorded to allow accurate scoring of the assessment once complete. This also allowed for the scores to be rated by an independent assessor to determine inter‐rater reliability. The participants were assessed in a quiet space familiar to the children, near the kindergarten classrooms. An occupational therapist skilled in administration of the DTVP (3) and the PWA (12), who was blinded to the aims of the intervention, completed these assessments. Inter‐rater reliability was determined on 20% of PWA (12) (*n* = 18). These measures were rescored by an independent rater who viewed the video recording as well as hard copies of the drawings the participants produced. In this study, the inter‐rater reliability of the expanded PWA (12) was established as excellent with an intra class correlation coefficient of 0.99 (*p* < 0.001).

#### 2.5.3 Fidelity checklist

A fidelity checklist using a dichotomous scale was purposely designed for this study based on established tools to assess treatment fidelity (Borrelli, 2011). This checklist addressed areas of study design, delivery of treatment, and receipt of treatment. The researcher completed this checklist at the end of each intervention session (*n* = 36), determining if an aspect of the intervention was either present or absent but should be present, achieving a total score out of 18. An independent assessor scored 20% of the sessions (*n* = 7), using the same checklist to determine inter‐rater reliability.

#### 2.5.4 Acceptability survey

A survey using a modified Likert scale was created to determine retrospective acceptability of the intervention, based on the Theoretical Framework of Acceptability (TFA) (Sekhon et al., 2017) and the Kids Intervention Profile (Eckert et al., 2017). This scale addressed acceptability areas of affective attitude, intervention coherence, perceived effectiveness, self‐efficacy, opportunity cost, and burden. An independent assessor facilitated the survey, so participants did not feel swayed to answer the questions in a certain way to the researcher who had delivered the treatment. The survey was administered the day following completion of the intervention, taking between 1 min, 44 seconds and 3 min, 42 s (standard deviation of 25.50 s) on site at the school in a room the participant was familiar with. A script was used by the independent assessor stating that the survey was not a test and that they would like to hear the participants' thoughts on the activities they had been doing. The participants were shown a supporting three‐point visual analogue scale and demonstrated they knew how to use the visual analogue with a practice question. The surveys were video recorded for later transcription, and the interviewer manually recorded the participant responses on a paper survey. Additional unprompted commentary from the participants was transcribed into a spreadsheet. An independent rater rescored 20% of these interviews (*n* = 4) to determine inter‐rater reliability.

### 2.6 Randomisation

A statistician assisted random allocation of the 18 participants to the intervention or waitlist control group. The participant names were placed into separate blank envelopes which were coded, and the participants were allocated using the odd and even roll of a die to intervention or waitlist control groups, until nine participants were allocated to both groups. The process was repeated to evenly allocate the participants into one of the three intervention groups or three waitlist control groups, with three participants in each group. Once allocated to the groups, a coding spreadsheet was established to ensure the researcher remained blinded to the participants outcomes. The participants and families were blinded to as to whether they were assigned to the intervention group or waitlist control group; however, teachers were informed so they were aware of when students would be leaving their class for the intervention.

### 2.7 Data analysis

A mixed ANOVA was used for this exploratory analysis to determine between group comparisons with Time (two levels: pre‐ and post‐intervention) as the within‐participant variable and Group as the between‐participants variable. Post hoc pairwise comparisons included Bonferroni adjustments for alpha values. A significant effect of Group × Time interaction was predicted. All analyses were conducted using SPSS Version 28. A significant main effect of Time was predicted. The dependent variable was the percentage of success on the PWA (12). The Reliable Change Index (RCI) was used to determine change in the CO and EHC subtests and VMI composite index of the DTVP (3). The RCI statistic calculates whether an individual's change in score (i.e. pre–post difference in standard scores is statistically significant by using the reliability values of a standardised test. The RCI is calculated using the formula *x*2 − *x*1/Sdiff, where *x*1 is the participant's pretest score, *x*2 is the same participant's post test score, and Sdiff is the standard error of difference between the two test scores.

Intraclass correlation coefficients, using absolute agreement and single measures in a one‐way random‐effects model, were used in SPSS to determine the inter‐rater reliability of the PWA (12). Excel software was used to calculate descriptive statistics for both the acceptability and fidelity checklists.

## 3 RESULTS

The Template for Intervention Description and Replication (TIDieR) guide and Consolidated Standards of Reporting Trials (CONSORT) were used to guide the reporting of this pilot. See supplementary information for completed TIDieR and CONSORT checklists.

### 3.1 Recruitment

**FIGURE 2:**
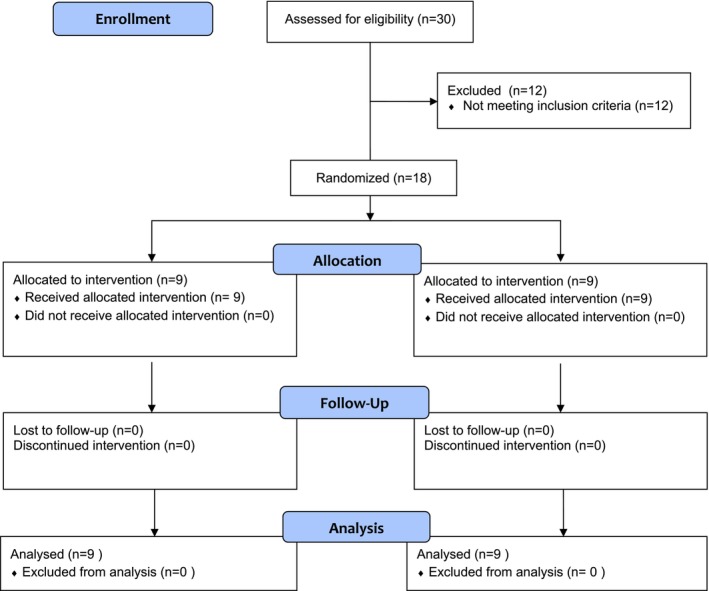
Recruitment flowchart.

The participants were recruited in May 2023 when teachers were asked to identify students that met the eligibility criteria. A total of 30 students were assessed for the second stage of eligibility prior to a final sample of 18 participants from across the three kindergarten classes being determined (7 = female, 11 = male). The intervention group included six females and three males, with a mean age of 4 years, 6 months (standard deviation of 3 months). The waitlist control group included eight males and one female with a mean age of 4 years, 4 months (standard deviation of 2 months). The mean VMI of the DTVP(3) for the intervention group was 104 (standard deviation of 9.6, median 106) compared to a mean of 99.67 (standard deviation 9.66, median 100) for the waitlist control group The mean percentage of success for the PWA(12) was 41.18 (standard deviation 10.19, median 47.60) for the intervention group and 32.89 (standard deviation 12.38, median 37.00) for the waitlist control group. Within the intervention group 88.89% (*n* = 8) improved their performance in the PWA following intervention. The waitlist control group saw that 100% (*n* = 9) improve their performance in the PWA(12) following the intervention. See Figure 2 for recruitment flowchart. See Table 3 for baseline and post intervention scores on the DTVP(3) and PWA.

**TABLE 3:** Baseline and post intervention scores on the DTVP(3) and prewriting assessment.

Group	Baseline scores				Post‐intervention scores			
	DTVP (3) VMI	DTVP (3) EHC	DTVP (3) CO	PWA (12)	DTVP (3) VMI	DTVP (3) EHC	DTVP (3) CO	PWA (12)
	*Composite index mean, median, standard deviation*	*Scaled score mean, median, standard deviation*	*Scaled score mean, median, standard deviation*	*Success (%)* *mean, median, standard deviation*	*Composite index mean, median, standard deviation*	*Scaled score mean, median, standard deviation*	*Scaled score mean, median, standard deviation*	*Success (%)* *mean, median, standard deviation*
Group 1	104	12.89	8.64	41.18	98.33	10.44	9	60.92
Intervention	106	14	8	47.60	100	11	9	66.33
*n = 9*	(9.60)	(2.20)	(1.67)	(10.19)	(6.20)	(1.59)	(1.73)	(17.68)
Group 2	99.67	11.00	8.89	32.89	94.33	9.67	8.67	55.52
Waitlist control	100	10	9	37.00	91	10	9	58.66
*n = 9*	(9.66)	(1.87)	(2.03)	(12.48)	(8.93)	(1.12)	(2.12)	(18.52)

*Note*: DTVP(3) = Developmental Test of Visual Perception Third Edition, VMI = visual motor integration, EHC = eye–hand coordination, CO = copying, PWA = Prewriting Assessment.

### 3.2 Primary outcome

#### 3.2.1 Treatment effect

There was a statistically significant main effect of time on the PWA (12), *F*(1, 16) = 12.79, *p* = 0.003, partial *η*_2_ = 0.444. Post hoc pairwise comparisons indicate that there was a statistically significant increase in prewriting ability (as determined by the percent of success in the PWA [12]) preintervention (*M* = 48.408, *SD* = 4.028, 95%CI [39.87, 56.95]) to post intervention (*M* = 53.758, *SD* = 4.150, 95%CI [44.96,62.56], *p* = 0.003). The main effect of group was not statistically significant, *F*(1,16) = 3.77, *p* = 0.070, partial *η*_2_ = 0.191. The Time × Group interaction was not statistically significant, *F*(1, 16) = 0.730, *p* = 0.406, *η*_2_ = 0.044.

While the VMI subtests of the DTVP3 were not used as an outcome measure in this study, it was identified that four of the 18 participants exceeded the Reliable Change Index (RCI > 1.96) in the CO subtest, indicating statistically significant improvement. No statistically significant change was observed in any of the participants on the EHC subtest or the overall VMI composite.

### 3.3 Ancillary analysis

#### 3.3.1 Feasibility

##### Recruitment and retention rate

Of the 30 participants screened in the study, 18 of these were deemed eligible to participate (60%). The participant's absence occurred across five of the 36 intervention sessions because of illness or vacation. For two participants who were unwell, they received an individual make up prior to their next group session. One other participant was absent for two subsequent sessions; however, they received a make‐up session prior to their next group session which addressed the content of both sessions missed. One other participant missed their final intervention session because of vacation which could not be replicated owing to timing of school holidays. In total, 17 of the 18 participants received six intervention sessions (94.44%), in sequence and before the collection of the post‐data time point. See Figure 2 for recruitment flowchart.

##### Data collection

All 18 participants completed every stage of assessment in pre and post testing using the PWA (12) and DTVP (3) (100%). Regarding acceptability, 17 of the 18 participants (94.44%) engaged in the one‐to‐one acceptability surveys and fidelity checklists were completed following every intervention session (*n* = 36, 100%).

##### Resources

Resourcing of this pilot study was feasible, with the lead researcher completing every intervention session and availability of a skilled independent occupational therapist to complete the data collection of the DTVP (3) and the PWA (12). The intervention in this pilot study was therefore deemed feasible in the context of delivering of a small group prewriting programme for 4‐ to 5‐year‐old children within a kindergarten setting.

#### 3.3.2 Fidelity

All group intervention sessions scored a total of either 17 or 18 out of possible 18 on the fidelity checklist. An independent rater rescored 20% of the checklists (*n* = 7), indicating 100% agreement. The group treatment sessions ranged from 18 min, 48 s to 37 min, 48 s, with a mean time of 27 min, 57 s and a standard deviation of 4 min, 47 s. The dosage criteria were not met because of participant's absence (less participants in the session), less content in early sessions (1 and 2), more content in later sessions (5 and 6), and the need for additional scaffolding to support engagement and manage behaviour in some sessions. See Table 4 for fidelity outcomes.

**TABLE 4:** Fidelity outcomes.

Area of fidelity	Total sessions meeting specific fidelity criteria	Total sessions not meeting specific fidelity criteria	
Study design			
Intervention protocol used	36	0	
Protocol has been written and printed for research student	36	0	
Resources are consistent for every group	36	0	
Training providers	N/A		
Delivery of treatment			
Dosage–session ran between 23 and 30 min	22	14	
Explicit introduction outlining the purpose of session	36	0	
Intervention protocol used	36	0	
Visual poster used			
Motor element where all students used their whole body	36	0	
Sensory element where all students used their hands and fingers in a tactile manner	36	0	
Tool‐based element where all students used chalk, whiteboard marker, and pencil/crayon	36	0	
Explicit check in at the end of the session	36	0	
Prewriting pattern drawn with pencil/crayon at least five times	36	0	
Verbal cue used at least 15 times	36	0	
Participant verbalises the rhyme at least five times	36	0	
Participant practiced the pattern at least 20 times among gross motor, tactile, and tools	36	0	
Receipt of treatment			
Check in on understanding of the content with each student at the end of session	36	0
Information presented in multiple formats; motor, tactile, verbal, and visual	36	0
Information presented in engaging way; multi modal with posters, rhyme tactile activity, and motor activity	36	0
Enactment of treatment	N/A		

*Note*: Training of providers was not relevant in this study as the treatment was provided by one researcher who designed and developed the intervention protocol.

Enactment of treatment was not evaluated as the ability to apply the learned information was captured by other outcomes measures that measured response to intervention over time, rather than within the one intervention session.

#### 3.3.3 Acceptability

Acceptability results indicate that participants ranked the sessions high for enjoyment, perceived effectiveness, and self‐efficacy. The participants also indicated that the intervention was hard. Four interviews were reviewed for inter‐rater agreement and 100% agreement was achieved. See Table 5 for acceptability outcomes.

**TABLE 5:** Acceptability outcomes.

Areas of acceptability	Ranking on scale			
	No	Yes *A little bit*	Yes *A medium bit*	Yes *A lot*
	*n*	*n*	*n*	*n*
Affective attitude				
Did you like drawing shapes with (name of occupational therapist) each week?	6		4	7
Do you like being told what to draw?	3	4	1	9
Were there times when you did not want to draw the shapes with (name of occupational therapist)?^a^	7	9	N/A	N/A
Intervention coherence				
Were there any times when you wished you could work on more drawing with (name of occupational therapist)?	4	13	N/A	N/A
Perceived effectiveness				
Do you think your drawing has improved? If yes, how much do you think your drawing has improved?	4	1	2	10
Self‐efficacy				
Are you good at drawing shapes. If yes, how good do you think you are at drawing shapes?^b^	2	1	2	11
Opportunity cost				
Did coming to the drawing with (name of occupational therapist) stop you from doing other things you wanted to do in class?	14	3	N/A	N/A
Burden				
Was it hard work drawing the shapes and doing the activities with (name of occupational therapist)?		7	N/A	N/A

*Note*: Questions indicating N/A are where a ranking scale did not apply.

^a^ One student did not answer.

^b^ One student was not asked the question.

## 4 DISCUSSION

This pilot study aimed to determine preliminary efficacy, feasibility, and acceptability of a prewriting intervention (Peggy Lego) when implemented with typically developing 4 to 5‐year‐old children. This study identified that while all participants improved their prewriting ability over time, there was no significant difference between participants who received intervention compared to those who did not receive intervention. Based on the findings of reliable change index however, some participants improved their CO skills following the intervention. Acceptability ratings indicated most participants felt their drawing improved following the intervention, and the treatment was implemented with high levels of fidelity.

Results showed that all participants improved over time with or without intervention, indicating either children naturally develop these skills or their classroom prewriting instruction or exposure was sufficient. Although in this study a school was sought that did not implement the Peggy Lego programme, the school did expose students to prewriting patterns as part of standard classroom practice in line with the Kindergarten Curriculum Guidelines, perhaps suggesting that this classroom exposure was sufficient in supporting the student's prewriting ability. Factors of teaching style, school attendance of children, Index of Community Socio‐Educational Advantage (ICSEA) of the school (Australian Curriculum Assessment and Reporting Authority, 2020), and existing skills and development of the children in the classrooms may all impact on the development of prewriting ability. Possible future trials of efficacy of the Peggy Lego programme would require rigorous collection of data relating to usual classroom prewriting teaching practice and exploration of these additional aspects to confirm if these factors impact on the prewriting intervention outcomes.

An important aspect of this pilot study was the inclusion of fidelity. Monitoring the treatment fidelity in such an early phase of research can improve future implementation as higher levels of treatment fidelity are associated with better treatment outcomes (Borrelli et al., 2005). The additional assessment of fidelity within this study determined that the intervention was implemented as intended. While the group analysis did not identify that small group intervention alone does not improve prewriting ability, we do know that the intervention was implemented as intended. In the absence of prewriting instruction programmes with established efficacy, the Peggy Lego programme can be replicated and implemented with high levels of fidelity, providing confidence that this programme can be replicated for further research and more likely to achieve intended treatment outcomes.

While treatment intensity is essential when planning intervention, there is limited research available relating to dosage and treatment intensity within early intervention (Warren et al., 2007), and further research related to dosage for handwriting interventions is required (Cole, 2022). For remediation of handwriting difficulties, at least 20 intervention sessions, twice per week, have been recommended (Hoy et al., 2011); however, another study on graphomotor skills in children indicated that 12 treatment sessions (with 15 repetitions per session) provided immediate and sustained improved performance compared to six or 24 treatment sessions (Ghanamah et al., 2020). Ghanamah et al. (2020) identified that it is likely that both the amount of practice within a treatment session and the schedule of the treatment can impact on the retention of the graphomotor skills. This pilot study included only six Peggy Lego treatment sessions (with 20 repetitions per treatment session), which allowed all prewriting patterns to be addressed in the treatment. Distributed practice was not provided, however, between the weekly treatment sessions, aside from a review within subsequent treatment sessions of prewriting patterns learnt in prior treatment sessions. This intensity of the Peggy Lego treatment and dosage may have been insufficient as participants may still have been within the cognitive or associative phase of motor learning and hence a dosage of six sessions may not be sufficient in supporting participants to develop automatic recall of the prewriting patterns. While it could be argued that distributed practice may have occurred (as it was likely participants were exposed to prewriting concepts in the classroom as part of standard classroom practice), this exposure did not involve direct practice of the Peggy Lego concepts and language. As such, the dosage, intensity, and distributed practice of the treatment in this pilot may have been a factor that impacted on the overall effect on prewriting ability from pre to post intervention.

Within this pilot study, the participants had to meet eligibility criteria and demonstrate average or above average VMI ability. Additional small group Peggy Lego instruction, however, may be beneficial for students who are not developing their prewriting ability alongside their peers or responding to general classroom exposure. This may include students with below average VMI. If this study was replicated with students who were not responding to classroom instruction or exposure or were not developing their prewriting ability at the same rate as their peers, then we may identify that small group intervention would be beneficial for this target population.

The inclusion of a survey to determine acceptability of the intervention from the participants perspective was unique as no evidence could be sourced regarding the acceptability of other prewriting or handwriting interventions. Ultimately, if an intervention is not acceptable to the participant, the likelihood of this intervention being effective in future trials (Sekhon et al., 2017) or in future classroom instruction is limited, hence, determining the acceptability can provide further justification for advancing the research. The creation of a purposely designed survey, based on the Theoretical Framework of Acceptability (Sekhon et al., 2017) and the Kids Intervention Profile (Eckert et al., 2017), with a supporting visual analogue scale, provided insight into a child's perspective on the acceptability of the Peggy Lego intervention. Given the high ranking of acceptability by the participants in terms of the enjoyment, perceived effectiveness and self‐efficacy along with the high rate of inter‐rater agreement of this survey, we can be confident that this intervention is likely to be acceptable to participants. Importantly, this pilot identified that participants perceived that their drawing ability improved following the treatment, even though most found it hard. These high levels of perceived effectiveness may support students as they move into more formal letter writing instruction when they commence full‐time schooling. It is at this next stage of handwriting instruction, formal letter writing, where handwriting becomes more complex with the added cognitive load of understanding the concepts of letters and sounds. As such, further research could explore if high levels of perceived self‐efficacy in prewriting supports students in mastering efficient letter formations.

Further studies could investigate if prewriting ability in the kindergarten year supports students transition to more formal handwriting instruction that focusses on the formation of letters. The task of forming a letter involves many component level skills, not just motor skills, and other factors that impact on letter formation, including orthographic knowledge need to be considered (van Galen, 1991). Monitoring of students handwriting development over time would have to include a broader assessment of the language elements of writing such as letter and sound awareness.

### 4.1 Limitations

The formation of letters involves a series of ‘rules’ regarding the directionality used to form the letter and the quality with which is it produced (Mathwin et al., 2022). Creative drawing, however, is not bound by the same ‘rules’ of directionality. The PWA (12) used in this study determined prewriting ability based on the replication of a drawing using prewriting patterns with set directionality. Even though there is evidence to indicate that drawing is strongly linked to handwriting ability (Steffani & Selvester, 2009), the unique and creative element of an individual's drawing may have impacted on the findings of this study.

Factors relating to the sample size may have impacted on the results. Low power increases risk of Type 2 error, and this study included a small sample. As such, this pilot study was underpowered to detect between‐group differences or rare adverse effects. With a larger sample size and increased power, we may also be able to determine the efficacy of this programme with greater confidence.

Progression criteria were not established as part of the planning for this study design. As such, if this study progresses to the next phase of research, official stop and go criteria would need to be embedded as part of the study design.

There is no psychometric evidence supporting the cut‐off ceiling score used for the PWA (12) in this study. For future studies, review of this ceiling score to determine participant eligibility would be required.

This study aimed to determine if small group intervention improved prewriting ability. While we identified that participants improved their prewriting ability irrespective of receiving intervention, we have not been able to track the long‐term impact of prewriting ability on future handwriting function and automaticity.

### 4.2 Recommendations for future research

Further research is required to determine the treatment efficacy of this prewriting intervention. Future research should also investigate if early prewriting ability predicts future handwriting automaticity and if explicit instruction is a predictive factor in prewriting ability or a protective factor in preventing future handwriting dysfunction. Studies could investigate the role of prewriting intervention for students who are not responding to classroom instruction and if self‐efficacy and confidence in prewriting ability supports students in their transition to formal letter writing.

## 5 CONCLUSION

Research has identified that prewriting ability is often overlooked in research and assumed in instruction (Reutzel et al., 2019), yet it is an essential developmental aspect of learning how to handwrite. Mastering prewriting patterns is a prerequisite to accurate and efficient letter formation, and while there are some programmes available to use in therapy and in classroom instruction, there is a lack of studies with the highest level of design that can be used to inform best practice for prewriting programmes in the classroom (Klein et al., 2021a).

While all participants in this study improved their prewriting ability over time, they did not have greater improvement in their prewriting ability following prewriting intervention; however, most participants reported that following intervention, they felt they improved their drawing skills and were confident in their drawing skills. In the absence of prewriting programmes with established efficacy, this pilot has identified that the Peggy Lego prewriting intervention is feasible and can be implemented with high levels of fidelity. The recruitment process, inclusion criteria, and use of the DTVP (3) and PWA (12) as outcome measures could be used in future trials. Changes would be required including collecting detailed information from classroom teachers regarding the prewriting instruction and practice they provide. Refining the dosage of the repetitions within the session and the number of intervention sessions provided, along with calculation of an appropriate sample size, would also need to be considered. The findings of this pilot study have identified that it is feasible to further investigate the efficacy of the Peggy Lego programme that would provide a valuable contribution to the emerging body of evidence relating to prewriting skills of children and its instruction.

## AUTHOR CONTRIBUTIONS

Berenice Johnston, Brooke Ryan, Megan Hatfield, Samuel Calder, and Mary Claessen contributed to the study conception and design. Material preparation, data collection, and analysis were performed by Berenice Johnston. Brooke Ryan, Megan Hatfield, Samuel Calder, and Mary Claessen all contributed to the data analysis. The first draft of the manuscript was written by Berenice Johnston, and all authors commented on versions of the manuscript. All authors read and approved the final manuscript.

## CONFLICT OF INTEREST STATEMENT

No potential conflicts of interest are reported by the authors.

## Data Availability

The data that support the findings of this study are available from the corresponding author upon reasonable request.
